# Supplementary material for: Excellent Performances of Composite Polymer Electrolytes with Porous Vinyl-Functionalized SiO2 Nanoparticles for Lithium Metal Batteries
Source: Polymers (Basel). 2021 Jul 27;13(15):2468. doi: 10.3390/polym13152468 (PMC8347765; doi:10.3390/polym13152468)
Supplement: Supplementary file 1 [file polymers-13-02468-s001.zip › polymers-1314611-supplementary.pdf]

polymers

MDPI

Supporting information

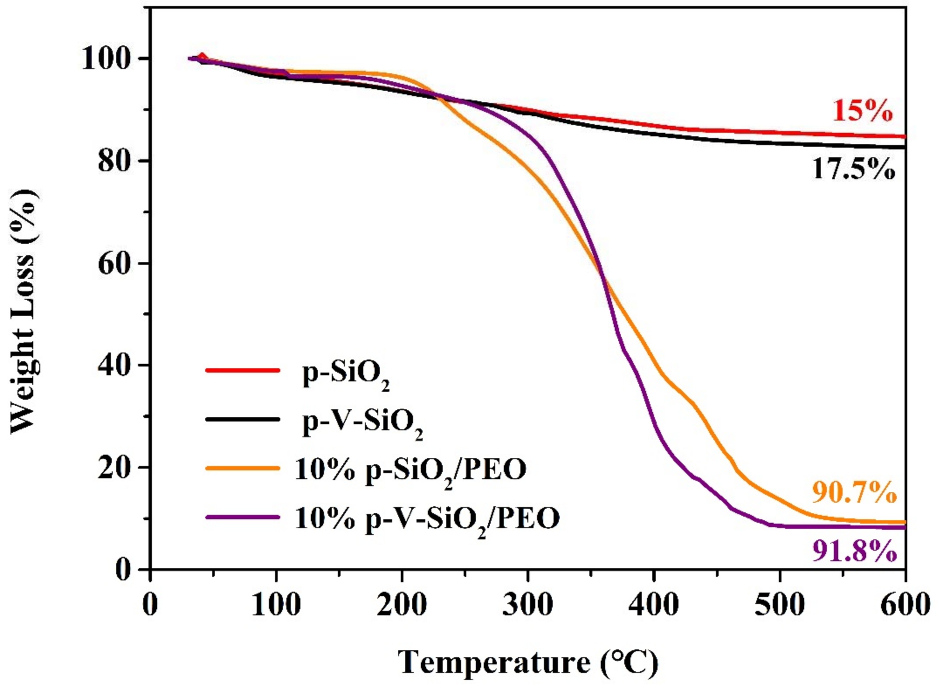

Figure S1. TGA data of p-SiO_2, p-V-SiO_2, 10% p-SiO_2/PEO and 10% p-V-SiO_2/PEO

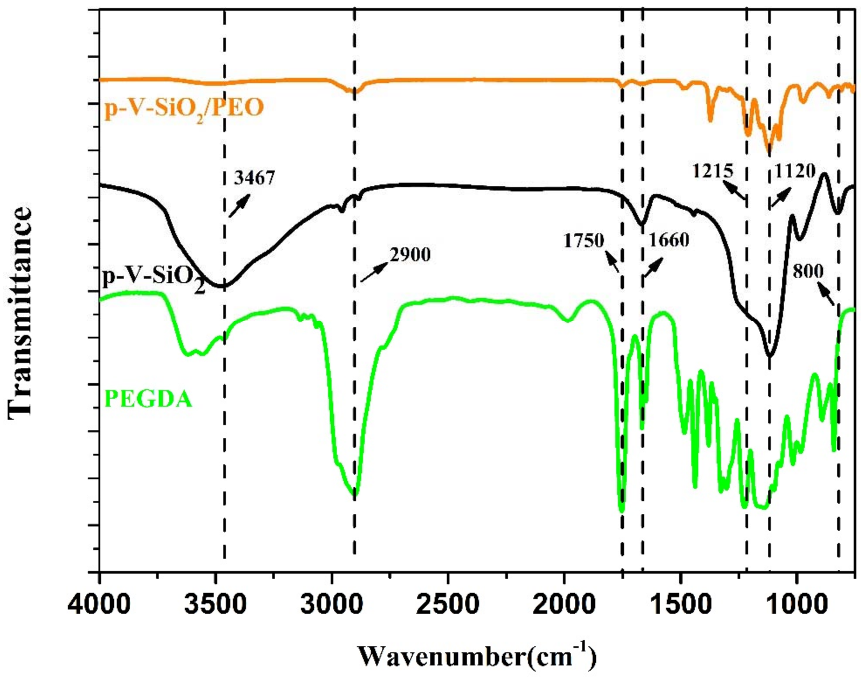

Figure S2. FT-IR spectra of PEGDA, p-V-SiO_2 and 10% p-V-SiO_2/PEO

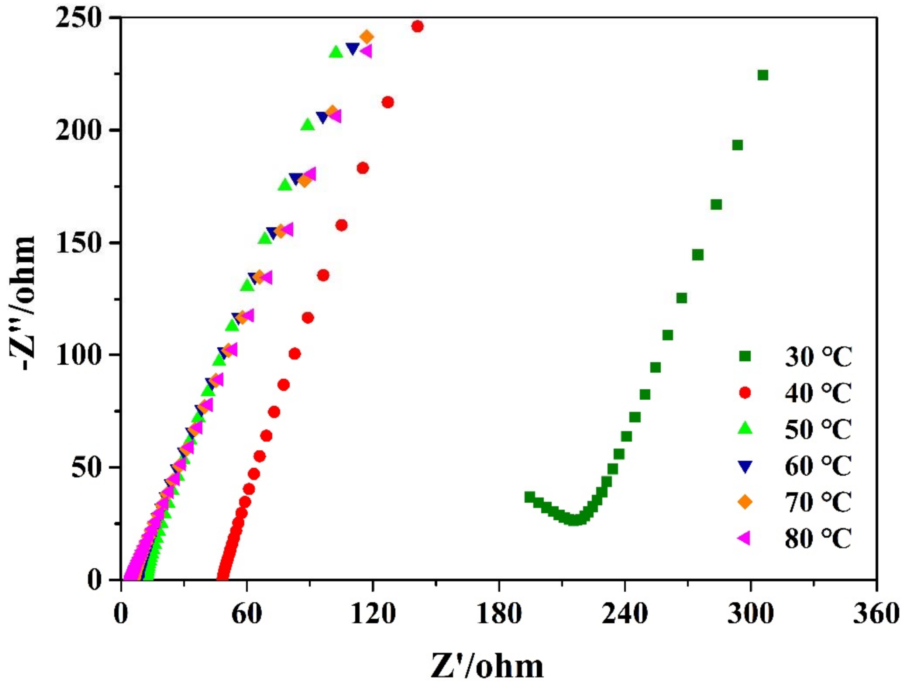

Figure S3. EIS profiles of 10% p-V-SiO_2/PEO CPE at the range of at the range of 30-80 °C.

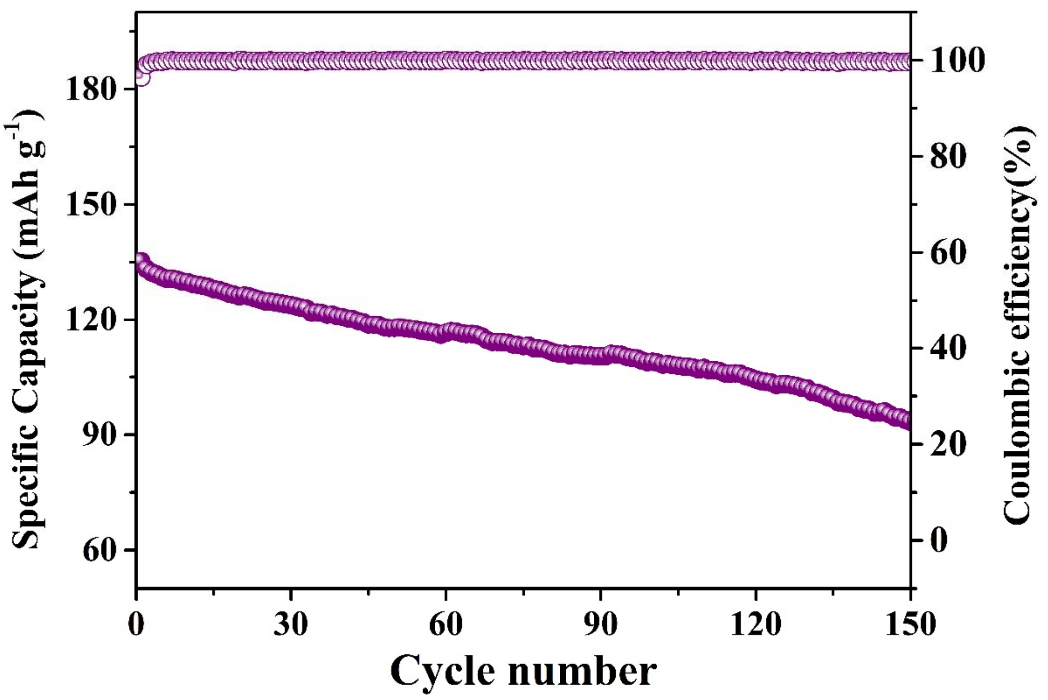

Figure S4: Cycle performance of Li|10% p-V-SiO_2/PEO CPE|LiFP cells at 1 C at 60 °C.
